# Totally tubeless single access tract mini-percutaneous nephrolithotripsy in treatment of large burden > 2-cm and/or complex renal stones: a case series of 62 patients

**DOI:** 10.1186/s12894-022-01012-9

**Published:** 2022-04-16

**Authors:** Chang-Heng Lin, Ying-Chen Lin, Heng-Chieh Chiang, Meng-Yi Yan, Wan-Yun Fang, Pao-Hwa Chen

**Affiliations:** 1 Division of General Practice, Department of Medical Education, Changhua Christian Hospital, Changhua, Taiwan; 2 Department of General Surgery, Division of Urology, Changhua Christian Hospital, 135, Nanxiao St., Changhua City, Changhua County 500 Taiwan; 3 Department of Chemical Engineering, Chung Yuan Christian University, Taoyuan, Taiwan

**Keywords:** Mini-perc, PCNL, Mini-PCNL, Single tract, 16 F

## Abstract

**Background:**

Limited literature has focused on the use of totally tubeless mini-percutaneous nephrolithotomy (PCNL) for the treatment of large renal stones. We present our findings of treating patients with large and/or complex renal stones using single renal access totally tubeless mini-PCNL.

**Methods:**

From March 2018 to May 2021, 62 consecutive cases in which single tract totally tubeless mini-PCNL was used to treat complex renal stones were enrolled, all with calculi > 2 cm. All procedure of puncture and dilation were guided by fluoroscope. The complexity of stones was assessed according to the Guy’s Scoring System (GSS). The surgical duration, length of hospital stay, analgesia requirement, stone-free rate, and perioperative morbidity were assessed.

**Results:**

The mean preoperative stone burden was 36.69 ± 19.76 mm (above 2 cm in all cases), mean surgical duration was 61.93 ± 40.84 min (range 15–180 min), and mean hematocrit reduction was 4.67 ± 2.83%. Postoperative Nalbuphine was used in 6 patients. The mean length of stay was 2.46 ± 1.19 days (range 2–8 days), and the postoperative stone-free rate was 83.9% (52/62), and 87.1% (54/62) after auxiliary ESWL. The overall complication rate was 14.5%, the majority of complications being postoperative transient fever.

**Conclusion:**

For the treatment of large bursen > 2 cm and/or complex renal stones, totally tubeless single tract mini-PCNL ensures a feasible SFR, low morbidity and short hospital stay. According to the low complication rate in our study, the totally tubeless manner was not associated with an increased risk of postoperative morbidity, and patients benefited from decreased postoperative analgesics use.

## Introduction

Urinary stone disease has affected humankind for centuries, with worldwide rises in incidence and prevalence in recent decades. The lifetime prevalence of nephrolithiasis in Asia is 1–5%, with a 50% recurrence rate within 5 years [1]. Urologists employ minimally-invasive endoscopic surgeries to maximize stone clearance and minimize complications.

Due to a higher stone clearance rate as compared with extracorporeal shockwave lithotripsy or retrograde intrarenal surgery, percutaneous nephrolithotomy (PCNL) is the first-line treatment for renal stones > 2 cm [2]. However, the better stone-free rate (SFR) of PCNL comes at the expense of greater risks and complications, such as blood loss or a longer length of stay (LOS), owing to its invasiveness [3]. As compared with standard PCNL, mini-PCNL (defined by a percutaneous tract ranging from 11 to 20 French, F, in diameter) carries a lower risk of complications while achieving a similar SFR. Cheng et al. [4] showed that mini-PCNL resulted in a better SFR for multiple calyceal or staghorn stones at the expense of a greater surgical duration, but resulted in fewer bleeding complications. However, the reported complications associated with mini-PCNL vary among studies. Bleeding complications requiring transfusion and/or arterial embolization in mini-PCNL studies were attributed to a larger stone burden, which required multiple renal access tracts [5]. Compared with standard PCNL, totally tubeless (without a double-J stent and nephrostomy tube) PCNL offers a reduced surgical duration, lesser hemoglobin change, shorter LOS and reduced demand for additional analgesics [6–8]. Several researchers demonstrated similar postoperative complication rates in tubeless PCNL and standard PCNL, which support tubeless PCNL as a safe and beneficial procedure in selected cases [7–9]. Even though studies have shown no statistically-significant difference in SFR between mini-PCNL and standard PCNL, mini-PCNL studies had lower stone burden as compared with standard PCNL [10, 11]. In cases of large and/or complex renal stones, urologists tend to choose standard PCNL to decrease the surgical duration.

Few studies have addressed totally tubeless mini-PCNL, especially for the treatment of large complex renal stones. In this retrospective study, we examined the outcomes and complications of treating large and/or complex renal stones using single renal access totally tubeless mini-PCNL.

## Methods

### Design and setting

The Urology Department at Changhua Christian Hospital (CCH) started performing PCNL in 1987, and currently average 150–200 PCNL procedures annually. After Institutional Review Board approval of the retrospective study (IRB number: 140315), 62 patients with complex renal stones (> 2 cm in length) were enrolled from March 2018 to May 2021. A single surgeon (PHC) performed totally tubeless mini-PCNL with a single 16F access tract. Stone complexity are graded according to the Guy’s Stone Score (GSS) preoperatively. The primary outcomes include SFR and postoperative morbidities.

### Preoperative preparation

Preoperative evaluation included complete blood work-up, urine analysis, urine culture and image studies, including kidneys ureter and bladder (KUB) X-ray, kidney ultrasound, intravenous urography and/or kidney computed tomography (CT). Patients with clinical evidence of urinary tract infection were treated with antibiotics for 4–7 days prior to surgery. Stone size burden was calculated by measuring the maximal length and width on the KUB X-ray or CT scan. If multiple stones were present, the total (cumulative) stone burden was documented as the sum of the length of each stone. All patients were admitted the day before surgery to ensure NPO (nil by mouth) status, and the LOS was calculated from the admission day to the discharge day.

### Surgical techniques

Prophylactic cephalosporines and tranexamic acid were prescribed 30 min pre-surgery; alternative antibiotics were used if the patient was allergic to cephalosporines, or if preoperative culture showed cephalosporine resistance. Under general anesthesia, ureterorenoscopy was first used to check for obstructions in ureter. A 5F retrograde-pyelography (RP) ureteral catheter was placed at the renal pelvis and a Foley’s catheter was inserted. The patient was then transferred to the prone position. All renal access was obtained under fluoroscopic guidance using the “eye of the needle” technique. The puncture site was chosen to maximize stone clearance, usually the calyx that gives a straight direct tract. After successful puncture, a 0.038-inch guidewire was introduced through the needle sheath into the collecting system. An Amplatz fascial dilator (Microvasive, Natick, MA, USA) was used to dilate the access tract to a diameter of 16F, and an outer sheath was placed into the targeted calyx. A 12F nephroscope (Richard Wolf GmbH, Knittlingen, Germany) and Holmium laser (12-W or 35-W) lithotripter were used for stone fragmentation. The stone fragments were either washed out or grasped through a zero-tip stone basket. An initial stone-free status was defined as no visible stone under direct vision, and was checked via intra-operative fluoroscopy. After the stone had been evacuated, a guidewire was placed into the collecting system and the access sheath slowly retracted. The access tract was carefully checked for severe bleeding, and fluoroscopy was employed to check along the RP catheter for any stone impaction.

### Postoperative care and outcomes

Operative findings, surgical duration (from insertion of the puncture needle to the end of the procedure) and outcomes were documented. The urine catheter was removed on postoperative day 1. All patients were prescribed 3-day Cefazolin and diclofenac (25 mg three times daily) or Acetaminophen (500 mg four times daily, for patients with impaired renal function) for prophylactic antibiotics and pain control. Intravenous 10-mg Nalbuphine was used for additional pain control when needed, and the overall dosage of intravenous analgesia was documented and analyzed. Complete blood work and biochemistry tests were performed on postoperative day 2. Renal ultrasonography, CXR and KUB were performed as routine before discharge to confirm the stone-free status and exclude the occurrence of hemothorax, urinoma or perirenal hematoma. Renal ultrasound and KUB were performed 1 month after surgery during an outpatient clinic visit. The initial stone-free status was checked at the end of the operation, and a final stone-free status was defined as a residual stone ≤ 2 mm under renal ultrasonography 3 months after PCNL. The Clavien-Dindo grading system was used to assess any surgical-related morbidity.

## Results

A total of 62 patients with large renal stones underwent single tract mini-PCNL (Table 1). The mean age of the patients was 58.82 ± 12.94 years. The average stone burden was 1172.66 mm^2^ with a maximum diameter of 36.69 mm. According to Guy’s scoring system (GSS), patients were stratified into 4 groups as shown in Table 2. Staghorn or partial staghorn stones accounted for 40% (25) of our cases. Primary outcomes including surgical duration, initial/final SFR, hematocrit and serum creatinine changes, and perioperative morbidities are listed in Table 3. All our patients were able to undergo a totally tubeless procedure.

**Table 1:** Patient demographic profile

	N (%)
Patients	62
Gender	
F	22 (35)
M	40 (65)
Age (years)	
Mean	58.82 ± 12.94
Range	34–83
Body Mass Index	26.18 ± 3.86
Laterality	
Left	26 (42)
Right	36 (58)
Stone location	
Staghorn or partial staghorn	25 (40)
Renal	21 (34)
UPJ or upper ureter	16 (26)
Stone burden	
Length (mm)	36.69 ± 19.76
Width (mm)	24.79 ± 14.86
Area (L × W) (mm^2^)	1172.66 ± 1693.89
Stone composition	
Whewellite or Weddellite	50 (81)
Struvite	10 (16)
Uric acid	2 (3)
Hydronephrosis	
Grade 1–2	44 (71)
Grade 3–5	18 (29)

**Table 2:** Classification of 62 patients according to the GSS

GSS	The patients stratified according to the Guy’s Stone Score	No. (%)
GS I	A solitary stone in the mid pole with normal anatomy	1 (2)
N = 21	A solitary stone in lower pole with normal anatomy	6 (10)
	A solitary stone in the renal pelvis with normal anatomy	14 (23)
GS II	Multiple stones in a patient with simple anatomy	12 (19)
N = 15	A solitary stone in a patient with abnormal anatomy	1 (2)
	A solitary stone in the upper pole	2 (3)
GS III	Partial stag horn calculus	21 (34)
N = 22	Stone in calyceal diverticulum	1 (2)
GS IV	A complete stag horn calculus	4 (6)
N = 4		

GSS: Guy’s Scoring System

GS: Guy score

**Table 3:** Outcomes and complications

	N (%)
Totally tubeless	62
Operation time (min)	61.93 ± 40.84
Post-operation	
LOS (days)	2.46 ± 1.19
Cr decrease (mg/dL)	0.05 ± 0.39
Hct decrease (%)	4.67 ± 2.83
Nalbuphine usage	6 (10)
Initial stone free (post OP)	52 (83.9)
Final stone free (3 months)	54 (87.1)
Clavien grading system	
None	53 (85)
Grade 1 (transient fever)	5 (8)
Grade 2 (sepsis, bleeding)	4 (6)
Grade 3 (second look, pneumothorax, blood clot causing urine retention)	0
Grade 4 (organ failure)	0
Grade 5 (death)	0

The mean surgical duration was 61.93 ± 40.84 min (range, 15–180). Longer surgeries were noted in cases of larger stones, which required more time for the fragmentation and extraction of stones. Nine cases had a surgical duration of over 100 min, the stone burdens for all 9 cases being above average, with 4 cases above 3000 mm^2^. The mean reduction in hematocrit percentage was 4.67 ± 2.83. Postoperative Nalbuphine was only used in 6 patients (mean dosage, 15 mg). The mean hospital LOS in the 62 cases was 2.46 ± 1.19 days (range, 2–8).

Transient fever, defined as a postoperative body temperature > 38.0 °C, which either self-resolved or resolved after antipyretic use was observed in 5 cases (8.1%). One case (1.6%) of sepsis was noted, which was controlled with a full course of antibiotics, and the patient was discharged uneventfully on postoperative day 8. Intra-operative bleeding occurred in 3 cases (4.8%); the bleeding did not interfere with the surgery and was related to collecting system perforation. Bleeders were controlled with adequate sheath placement assisted by a bleeding tamponade. No patient required a blood transfusion or arterial embolization, and no peri-renal hematoma or fluid accumulation was found during follow-up. No other complications, such as pneumothorax, injury to adjacent organs, urine extravasation, or mortality, occurred.

An initial stone-free status was achieved in 52 patients (83.9%). Seven patients required auxiliary ESWL in the postoperative setting, which resulted in a final SFR of 87.1% after the auxiliary procedure. Stone chemical analysis revealed calcium oxalate as the chief component (Whewellite or Weddellite) in 50 cases (81%), struvite in 10 (16%), and uric acid stone in 2 cases (3%).

## Discussion

Data regarding the benefits of the minimal invasiveness of mini-PCNL as compared with standard PCNL vary among the English literature. Several studies failed to demonstrate a benefit of a smaller nephrostomy tract in preventing renal parenchymal damage [12, 13]. However, studies have shown mini-PCNL to result in a higher stone clearance rate, shorter hospital stay, lower transfusion rate and fewer morbidities as compared with standard PCNL. Several researchers have also stated that complex renal stones are amenable to mini-PCNL without resulting in increased complications, regardless of stone burden. In order to obtain a similar SFR with a larger stone burden, mini-PCNL requires a longer surgical duration than standard PCNL [4, 12, 14, 15]. Factors contributing to the longer surgical duration when treating complex stones with mini-PCNL included use of a laser for fragmentation/dusting, and multiple access tracts, which increase the risk of bleeding resulting in an impaired operative field.

The employment of laser lithotripsy equipment can greatly affect the surgical duration. Larger stone fragments are obtained with a larger-diameter laser fiber [16]. In our series, we used a 200-μm diameter paired with 12-W or 35-W lasers. We envisage that using the newer 60 − 120-W laser lithotripsy machines would reduce the stone disintegration time by half.

In our study, single access tract mini-PCNL was performed in cases of complex renal stones > 2 cm, totaling 25 cases (40%). The average surgical duration and bleeding complications were similar to those reported in the global CROES study, and no patients required blood transfusion or embolization [17]. In addition to a single access tract, the minimal bleeding complications in our study can be attributed to careful selection of the calyx and puncture angle. Puncture was made through the avascular plane of Brödel in a minor calyx that resulted in an access tract as close to parallel to the infundibulum as possible to minimize the bending angle and reduce the risk of injury to the interlobal artery and parenchyma.

Factors that affected the SFR in this study included huge burden and complexity of stones (42% above Guy score III), a rigid nephroscope, and a single nephrostomy tract [18–20]. Despite our SFR being similar to those reported in other mini-PCNL studies, most similar studies usually report a stone burden less than 2 cm, and the stones are not usually complex [10, 21]. Our initial SFR of 83.9% and postoperative SFR at 3 months of 87.1% after auxiliary procedures were also on par with those reported in the global CROES study for staghorn stones, and were better than the reported SFR in studies related to complex renal stones [18, 22, 23].

Different exit strategies after PCNL have been evaluated in recent years to reduce postoperative pain, LOS, and complication rate. Istanbulluoglu et al. and Nalbant et al. reported that avoiding the use of a nephrostomy tube and double-J stent resulted in less postoperative pain and consequently less need for analgesics as compared with patients undergoing standard PCNL [24, 25]. In addition, advantages of the totally tubeless procedure over the standard one were noted in terms of a shorter surgical duration and hospital stay [6, 24–26]. In our study, six patients (9.7%) required intravenous analgesics, and the average LOS of all patients was 2.5 days, without serious complications. Due to calculation of LOS including the preoperative survey and the day of surgery, the mean LOS was only approximately 1.5 days. Placement of a nephrostomy is helpful for tamponade during severe bleeding, assisting renal healing and avoiding urine extravasation. In complex renal stone cases, the need for a nephrostomy tube after PCNL remains under debate.

Moosanejad et al. [6] compared totally tubeless and standard PCNL in 84 patients in an RCT trial that included 12 staghorn cases (7 tubeless vs. 5 standard), and there were no statistically-significant differences between the two groups. Wang et al. [27] compared staged and simultaneous bilateral tubeless PCNL in 99 patients, and found that tubeless PCNL was associated with a low morbidity, short LOS, high stone-free rate, and early return to normal activity in cases of staghorn stones. In their meta-analysis, Lee et al. [7] noted that hemoglobin change and LOS were superior in totally tubeless and tubeless PCNL cases. Our series echoed the previously-reported safety and efficacy of single tract totally tubeless mini-PCNL for the treatment of large complex renal stones.

Fever and bleeding are two main perioperative complications of PCNL. In our series, the overall complication rate was 14.5% (9 of 62 patients), with transient fever being the most common. No Clavien grade III to V complications were noted (Table 3). In recent systemic reviews, the overall complication rate for mini-PCNL was reported to be 11.9–37.9% (Clavien grade I: 2.7–20.8%, II: 1.4–17.3%, III: 0–10.3%, IV: 0–0.05%, and V: 0–0.02%) and 15.2% (Clavien grade I: 44%, II: 28%, III: 28%).^10,11^ As per guideline suggestions, our protocol of preoperative urine culture along with a full course of oral antibiotics for 4–7 days appeared helpful in managing postoperative infection. A high intrapelvic pressure (IPP) causing pyelovenous backflow resulting in bacterial infection has always been thought to be a culprit for infectious complications post-PCNL. Wang et al. [28] showed microscopic pathological change in an animal model mimicking obstructive kidney reaching a pressure > 20 mmHg. In clinical practice, a lower “ratio of endoscope and sheath diameter” (RESD) could ensure a higher flow rate through the interspace between the nephroscope and renal access sheath, resulting in a decreased intrarenal pressure, and at the same time facilitating removal of stone fragments. Doizi et al. [29] enrolled cases treated by mini-PCNL with different working sheath sizes (range, 16–22F) and a fixed 12F nephroscope, and found a negative correlation between intrapelvic pressure and the diameter of the operating sheath. Fang et al. [30] tested different flexible ureteroscopes with differing ureteral access sheath diameters, and noted that a RESD lower than 0.75 resulted in a low IPP (< 13 cmH_2_O). Although we do not have an intra-renal pressure-measuring device at our hospital, our low infectious complication rate indirectly reflected a low IPP due to a low RESD of 0.75 (12F nephroscope and 16F access sheath). Limitations of our study included a non-randomized controlled nature and a small study population. Further studies randomizing patients with complex renal stones into groups of totally tubeless or tubeless, single or multiple renal access, and mini or standard PCNL may provide conclusions with a valid statistical power.

## Conclusions

For the treatment of large burden > 2 cm and/or complex renal stones, totally tubeless single tract mini-PCNL ensured a feasible SFR, low morbidity and short hospital stay. From the low complication rate in our study, the totally tubeless manner was not associated with an increased risk of postoperative morbidity, and patients benefitted from decreased postoperative analgesics use.

## Data Availability

The datasets for this article are available in the Changhua Christian Hospital (Changhua City, Taiwan) Medical Records Room data base repository. Due to privacy and ethical concerns, Since Changhua Christian Hospital (Changhua City, Taiwan) Medical Records Room data base repository not open to public access, the datasets generated during and analyzed during the current study are not publicly available but are available from the corresponding author upon reasonable request.
